# 3D Cultivation Techniques for Primary Human Hepatocytes

**DOI:** 10.3390/microarrays4010064

**Published:** 2015-02-16

**Authors:** Anastasia Bachmann, Matthias Moll, Eric Gottwald, Cordula Nies, Roman Zantl, Helga Wagner, Britta Burkhardt, Juan J. Martínez Sánchez, Ruth Ladurner, Wolfgang Thasler, Georg Damm, Andreas K. Nussler

**Affiliations:** 1 BG Trauma Center, Siegfried Weller Institut, Eberhard Karls University Tübingen, Schnarrenbergstr. 95, 72076 Tü̈bingen, Germany; E-Mails: anastasia.bachmann@gmail.com (A.B.); matthias.moll@student.uni-tuebingen.de (M.M.); britta.burkhardt@gmx.de (B.B.); jjmartinezsanchez@live.com (J.J.M.S.); 2 Institute for Biological Interfaces, Karlsruhe Institute of Technology, POB 3640, 76021 Karlsruhe, Germany; E-Mails: eric.gottwald@kit.edu (E.G.); cordula.nies@kit.edu (C.N.); 3 ibidi GmbH, Am Klopferspitz 19, 82152 Martinsried, Germany; E-Mails: rzantl@ibidi.de (R.Z.); hwagner@ibidi.de (H.W.); 4 Clinic for General, Visceral and Transplantation Surgery, Eberhard Karls University Tübingen, Hoppe-Seyler-Str. 3, 72076 Tübingen, Germany; E-Mail: ruth.ladurner@med.uni-tuebingen.de; 5 Department of Surgery, Ludwig-Maximilians-University of Munich Hospital Grosshadern, 81377 Munich, Germany; E-Mail: wolfgang.thasler@med.uni-muenchen.de; 6 Department for General, Visceral and Transplantation Surgery, Charité Medical University Berlin, Augustenburger Platz 1, 13353 Berlin, Germany; E-Mail: georg.damm@charite.de

**Keywords:** primary human hepatocytes, three-dimensional (3D) cell culture, two-dimensional (2D) cell culture, *in vitro* model, hydrogels, scaffolds, drug-induced hepatotoxicity, long-term culture

## Abstract

One of the main challenges in drug development is the prediction of *in vivo* toxicity based on *in vitro* data. The standard cultivation system for primary human hepatocytes is based on monolayer cultures, even if it is known that these conditions result in a loss of hepatocyte morphology and of liver-specific functions, such as drug-metabolizing enzymes and transporters. As it has been demonstrated that hepatocytes embedded between two sheets of collagen maintain their function, various hydrogels and scaffolds for the 3D cultivation of hepatocytes have been developed. To further improve or maintain hepatic functions, 3D cultivation has been combined with perfusion. In this manuscript, we discuss the benefits and drawbacks of different 3D microfluidic devices. For most systems that are currently available, the main issues are the requirement of large cell numbers, the low throughput, and expensive equipment, which render these devices unattractive for research and the drug-developing industry. A higher acceptance of these devices could be achieved by their simplification and their compatibility with high-throughput, as both aspects are of major importance for a user-friendly device.

## 1. Introduction

One of the main challenges in drug development and safety assessments is the prediction of *in vivo* drug-induced hepatotoxicity based on *in vitro* data. The number of laboratory animals in preclinical stages could be significantly reduced by cultivation systems that have been validated and approved by regulatory authorities. Nowadays the gold standard consinsts of primary human hepatocytes (pHH) in conventional monolayer cultures (2D), as hepatic functions and drug-metabolizing enzymes in freshly isolated hepatocytes are close to the *in vivo* situation [1,2]. However, the detection of metabolism-mediated hepatotoxicity of drugs fails *in vitro* [1,2,3], as hepatocytes rapidly lose their morphology [4,5] and their liver specific functions, such as detoxification, activity of phase I and phase II enzymes, and the production of plasma proteins like albumin [6,7,8] under these conditions. In patients, metabolism-mediated hepatotoxicity can lead to drug-induced liver injury (DILI), a severe clinical event associated with acute liver disease and liver failure [9,10]. Furthermore DILI is the main reason for post-market retraction and issuance of warnings of approved drugs as well as drug failure during clinical trials [11]. In a recent European retrospective study the incidence of DILI has been estimated to be ~19 new cases per 100,000 persons each year [12]. The poor predictability of DILI from preclinical animal experiments is based on species-specific differences in drug metabolism between experimental species and humans [13,14]. Therefore detection of hepatotoxicity before drugs are tested in animals and clinical trials is an important issue in order to minimize the occurrence of DILI. Consequently, there is a demand in the pharmaceutical industry for improved hepatocyte-based screening models reflecting human *in vivo* toxicity.

In this short review, we compare existing cultivation techniques for hepatocytes. We specifically focus on the latest advances in the cultivation of hepatocytes in a 3D environment and their application in preclinical drug development, including our own attempts to develop a microfluidic device.

## 2. 2D Cultivation Models for Hepatocytes

The advantages and drawbacks of hepatocytes cultured in different conditions are summarized in Table 1. The gold standard consists of primary human hepatocytes cultivated under monolayer conditions, as it has been demonstrated that the extrapolation of hepatotoxic data from other experimental species to humans is difficult due to interspecies differences [13,14]. 

**Table 1:** Advantages and drawbacks of cultivation systems for hepatocytes.

Cultivation	Advantages	Disadvantages	Reference
**2D**			
Monolayer	Gold standard for drug metabolism and toxicity	Rapid loss of morphology and cell polarity	[8,9,15]
	Ideal for testing interindividual and interspecies differences in metabolism	Rapid loss of drug metabolizing capability, decrease of albumin production and cell-cell interaction)	
	Maintenance of key functions as carbohydrate metabolism and plasma protein synthesis (24–72 h)	Limited availability	
Co-culture	Improved functionality of all cell types	No real standard established	[8,16,17,18,19]
	Increased expression of phase I and phase II enzymes	High variability between different laboratories	
	Maintenance of cell morphology		
	Inducibility of CYPs		
**3D**			
Hydrogels, scaffolds scaffold-free	Long-term maintenance of liver-specific functions	Lack of established standards	[8,9,15,20,21,22,23,24,25]
	Increased sensitivity towards drugs	Not adjusted to high throughput	
	Long-term expression of phase I and phase II enzymes	Cell recovery for further analysis is difficult	
Co-culture with liver-drived cell types	Improved expression of phase I and phase II enzymes, Including inducibility by drugs	No real standard established	[17,26,27]
		Not adjusted to high throughput, high variability regarding cell viability and differentiation	
	Maintenance of cell polarity, cell-cell contacts and bile canaliculi		
	Mimicks *in vitro* architecture		
	Longer cell viability		
Microfluidic devices	Sustained liver like cell functionality and increased liver specific functions	No standardized system available so far	[8,9,15]
		Not adjusted to high throughput	
	Precisely adjusted flow/drug concentrations		
	Enable microscopic examination		
	Formation of a sinusoid-like shape (HepaChip^®^)		
	Fast differentiation of the cells after flow induction		

As there exists an immense demand in the pharmaceutical industry for human hepatocytes for toxicological testing and as the existing cultivation techniques do not sufficiently mimic the *in vivo* situation, an improvement and standardization of the existing cultivation techniques are urgently required.

### 2.1. Heptocyte Monolayer Cultivation

Most systems for the cultivation of hepatocytes are 2D monolayer cultures, which are based on the coating of surfaces with extracellular matrices (ECM) such as collagen. Under these conditions, human hepatocytes maintain key specific functions such as carbohydrate metabolism, plasma protein synthesis and CYP expression for a few days [8,11,15,28]. The cultivation of hepatocytes under monolayer conditions has been extensively used for drug-testing, as well as for investigating protective drug effects and underlying mechanisms of drug toxicity [14,29,30]. Unfortunately, under these conditions, hepatocytes de-differentiate and lose their biotransformation capacity within a few days [28], conferring human hepatocytes a very limited time-frame for toxicological studies [8,9,29].

### 2.2. Co-Cultivation of Hepatocytes

One main obstacle of hepatocytes monolayer cultures in 2D is the lack of cell-cell interaction between individual hepatocytes and their limited ability to reproduce hepatotoxic effects *in vitro* [16]. Furthermore, current techniques for hepatocyte cultivation lack cell-cell communication between different cell types [8,16]. Co-culture of hepatocytes with different cell types such as human adipose-derived stem cells [17,27], human umbilical vein endothelial cells [26], fibroblasts [19], and different non-parenchymal liver cells [8,16] maintain cell functionality of all cell types and the biotransformation capacity of hepatocytes. However, these co-cultivation methods have a high lab-to-lab variability and do not match the requirements of the pharmaceutical industry, as they are not adjusted to high throughput so far.

### 2.3. 3D Cultivation Systems for Hepatocytes

In the human body, each tissue has a defined stiffness, and the cells are embedded in a three-dimensional environment which is not reflected under 2D cultivation conditions [31,32]. Although, for a long time, monolayer cultures have been a cost-effective and practical method to propel the investigation of hepatotoxicity, there exists an urgent demand for the development of systems which enable 3D cultivation of hepatocytes, which are in addition suitable for routine applications and high throughput screenings for drug development. An indispensable prerequisite for three-dimensional matrices is permeability to support metabolic activities, transport of large macromolecules and to enable cell-cell interactions [33]. As the maintenance of hepatocyte function and the delay of de‑differentiation are especially important during drug screening processes, several cultivation systems, which allow the assembly into three-dimensional (3D) structures, have been developed in recent years by using either extracellular matrix (ECM) proteins or synthetic scaffolds (reviewed in [34]). The advantages and drawbacks of 3D hepatic cell cultures are summarized in Table 1.

The interaction of hepatocytes with a three-dimensional environment preserves their function as well as the expression of specific transporters, cell-cell communication via tight and gap junctions, and the formation of bile canaliculi [8,35]. Although some of the commercially available 3D cultivation systems have already delivered promising data [36,37,38], the exclusion of a continuous flow which is known to facilitate active transport of nutrients, metabolites, and oxygen, is a major drawback [39,40]. However, the combination of 3D cultivation with a continuous flow often leads to complex systems with restricted throughput and the requirement of high cell numbers, which is in contrast to the limited availability of primary human hepatocytes. To overcome these restrictions, recent attempts have led to the development of miniaturized 3D flow culture systems in order to reduce the amount of required cells and to enable high throughput applications, as these features would facilitate acute and dose repeated hepatotoxicity screenings.

One of the most important aspects driving the investigation of matrices for the 3D cultivation of hepatocytes is the preservation of function, in particular of the activity of drug-metabolizing enzymes and of hepatic transporters, over several days (acute model) or even up to 4 weeks (chronic hepatotoxicity). Currently available systems can be classified into the three following categories (i) hydrogels, (ii) microporous scaffolds, and (iii) scaffold-free 3D formats, such as spheroids or cell aggregates.

#### 2.3.1. Hydrogels

Hydrogels present water-swollen polymers derived from natural or synthetic resources (Table 2). Naturally-derived hydrogels, such as collagen and Matrigel^®^, are composed of extracellular matrix and other proteins. Collagen can be easily extracted from rat tail tendons and used for the cultivation of cells between two layers of collagen (collagen-sandwich) [5], resembling the *in vivo* phenotype such as the polygonal shape, restoration of cell-cell contacts, formation of bile canaliculi and maintenance of liver-specific functions (CYP activities, albumin, and urea synthesis) [2]. Moreover, Matrigel^®^ is a mixture of matrix proteins secreted by Engelbreth-Holm-Swarm sarcoma cells, which seem to be superior to collagen, as overlayed hepatocytes exhibit an improved bile canalicular network formation [38] and preserved liver-specific functions [2].

However, although naturally derived 3D cultivation systems dispose of a high biocompatibility, the remaining obstacles are high batch-to-batch variations, impeding the reproducibility of experimental results, and poor mass transfer of nutrients and oxygen. In order to overcome these disadvantages, synthetically derived hydrogels have been developed. One of the most extensively used synthetic hydrogels is polyethylene glycol (PEG) as its hydroxyl groups can be easily modified by functional groups or attached to other molecules or bioactive agents [33,41]. The embedding of hepatocytes into PEG hydrogels supported consistent urea synthesis and CYP3A4 activity in comparison to the 2D culture, while the combination with poly(lactic-co-glycolic) acid (PLGA) nanoparticles further improved hepatocyte function [33]. The combination of PEG hydrogels with abundant biomaterial from the liver or peptides that are required for the cell-ECM interaction, such as heparin or the Arg-Gly-Asp (RGD), resulted in significantly higher levels of albumin and urea synthesis in comparison to cells cultivated in the hydrogel alone [42,43]. One commercially available peptide hydrogel with self-assembling properties is PuraMatrix™ (3D Matrix Inc, Waltham, MA, USA). So far, the main disadvantages of these hydrogels are the high costs for the adjustment to high-throughput analysis and the low number of available data on hepatocytes [44,45].

**Table 2:** Overview of static 3D cultivation systems for hepatocytes.

3D System	Source	Cell Type	Observation	Reference
**Hydrogels**				
Natural	Collagen	Human hepatocytes	Sensitivity to acetaminophen-induced intoxication	[3]
	Matrigel	Human Hepatocytes	Enhanced formation of bile canalicular networks	[38]
Synthetic	PEG		Functional groups can be attached to macromolecules	[41]
	+PGLA	Human hepatocytes	Consistent urea synthesis and increased CYP3A4 activity	[33]
	+Heparin	Rat hepatocytes	Stable urea and albumin synthesis for more than 3 weeks	[42]
	+RGD	Rat hepatocytes	Spheroid formation, maintenance of urea and albumin production for more than 4 weeks	[43]
	PuraMatrix™	Rat hepatocytes	Higher albumin and urea levels for up to 3 weeks	[44]
**Scaffolds**				
Natural	Chitosan		Provision of adhesion for hepatocytes	[46]
	+Heparin/alginate		Increased albumin synthesis	[47]
	+GHA		Increased albumin secretion and urea synthesis	[48]
	Alginate	Rat hepatocytes	Cell-cell and cell-extracellular matrix (ECM) interactions, phase I and phase II activity stable for one week, high urea and albumin synthesis	[49]
Synthetic	PVA	Rat hepatocytes	Urea synthesis maintained over 5 days	[50]
	PLA	Rat hepatocytes	Maintenance of albumin and urea synthesis as well as CYP1A and UGT-activity	[51]
	PS	Rat hepatocytes	Higher activity of CYP1A2, CYP2B1, and CYP3A2	[52]
		Human hepatocytes	Higher activity of CYP2B6 and CYP3A4	[37]
**Scaffold-free**				
	Spheroids	Human hepatocytes	Metabolism of lamotrigine and salbutamol	[53]
	Nanoculture plate	HepG2	Enhanced expression of albumin, CYPs and liver-enriched transcription factors (HNF4-α and C/EBPα)	[54]

#### 2.3.2. Scaffolds

In order to overcome the above-mentioned pitfalls of hydrogels, porous scaffolds have been developed in recent years. Besides the cell-ECM interaction, the defined pore size of the scaffolds, which is achieved during fabrication, enables the establishment of cell-cell contacts which are essential for the maintenance of hepatic function [55]. Naturally derived scaffolds are either based on a single macromolecular carrier, such as chitin, alginate, chitosan, and gelatin, or on mixtures of these macromolecules [46,49]. The cultivation of hepatocytes on alginate scaffolds has revealed higher urea and albumin levels for up to one week, while CYP activities and conjugating phase II reactions remained stable [49]. Similar results have been obtained for the duration of 15 days by using a chitosan-gelatin or a chitosan scaffold in combination with alginate or heparin [46,56]. The main advantage of these naturally derived scaffolds consists in their biocompatibility that is, at the same time, their major drawback, as it may cause batch-to-batch variability.

Attractive alternatives are synthetically derived polymer scaffolds with a high biocompatibility, such as scaffolds based on poly lactic acid (PLA), poly glycolic acid (PGA), or poly lactic-co-glycolic acids (PGLA), which were initially developed for tissue engineering and hepatocyte transplantation due to their biodegradability [57], or scaffolds based on polyvinyl alcohol (PVA) and polystyrene. Depending on the manufacturing process, these polymer scaffolds shape membranes, sponges or foam disks, which are all suitable for perfusion. The cultivation of hepatocytes on PVA scaffolds has led to the preservation of hepatocyte parameters, such as urea and albumin synthesis, for up to 5 days [50], while on PLA-based scaffolds, the activity of CYP1A and glucoronysl transferase has been kept stable for up to 15 days [51]. A comparable surface to traditional 2D culture is offered by polystyrene (PS) scaffolds, which are commercially available under the name Alvetex^®^ (Reinnervate Ltd., Durham, UK). These scaffolds led to a higher activity of CYP1A2, CAP2B1, and CYP3A2 in rat hepatocytes, while CYP2B6 and CYP3A4 maintained their enzymatic activity in human hepatocytes [37,52]. Another commercially available system is the Mimetix^®^ system from the Electrospinning Company Ltd (Didcot, UK), which is based on polymer poly-L-lactide (PLLA) fibers. HepB2 cells cultivated for up to 28 days on these scaffolds exhibited a higher CYP activity as well as an increased albumin synthesis and detoxification [58]). However, the collaboration between Electrospinning and Medicyte may soon deliver first results from hepatocytes cultivated on Mimetix^®^ scaffolds [59].

#### 2.3.3. Scaffold-Free Cultivation

The scaffold-free 3D cultivation of hepatocytes achieved a higher compliance through simplification and commercialization by different companies. The most critical point is the spheroid size, as spheroids which are larger than 200–300 μm are at risk of having necrotic areas, as oxygen diffusion is the main limiting parameter The approach of Scivax Inc. (Woburn, MA, USA) enables spheroid formation on nanoculture plates without the requirement of any scaffold. Spheroids generated from HepG2 cells displayed a higher expression of albumin, CYPs, and the hepatocyte-enriched transcription factors HNF4-α and C/EBPα [54]. The technique of the InSphero AG (Schlieren, Switzerland) enables the creation of self-assembling micro-tissues via the hanging-drop culture [36]. Reconstruction of micro-livers was enabled by generating spheroids of hepatocytes co-cultured with different types of non-parenchymal liver cells, like e.g., endothelial cells, Kupffer cells or macrophages. The generated micro-livers exerted hepatic functions, such as bile canaliculi formation and glycogen storage, while in long-term culture for up to 14 days, the micro-tissues showed an increased sensitivity towards repeated doses of diclofenac [36] and nanomaterials [60]. A further improvement of this method will be achieved by combining hepatocytes and non-parenchymal liver cells with defined cell numbers from the same donor. An important step in this direction was recently accomplished by a protocol enabling the isolation of parenchymal and non-parenchymal liver cells from the same donor [16].

In summary, it has become evident that the three-dimensional cultivation techniques are superior to the standard cultivation conditions in 2D, as hepatocyte function is maintained by the different hydrogels and scaffolds. However, a clear comparison between the different cultivation techniques is difficult, as the design of the individual studies is heterogeneous and direct comparisons with established cultivation techniques, such as other devices and/or collagen sandwich or 2D cultures, have been limited in these studies.

## 3. 3D Microfluidic Cultivation Systems for Primary Hepatocytes

In order to create a more physiological environment for hepatocytes and to combat poor oxygen and nutrient diffusion, different perfusion systems have been developed. The main objectives of these efforts is the development of a fully functional cell culture model mimicking the complex *in vivo* architecture of a liver lobule which is suitable for toxicological and pharmacological drug screening and research. One of the main issues during the development of these devices is the balance between flow and shear stress applied to hepatocytes, as they are extremely sensitive to oxygen concentrations with a high metabolic demand, while they are also vulnerable to shear stress exceeding 0.03 Pa [61]. The viability of hepatocyte cultures under high shear is lower than for cultures under 2D conditions [62,63].

First attempts resulted in large-scale bioreactors that depended on high cell numbers and therefore caused high costs, both aspects prevented their routine application in research and drug development, while more recent developments focus on the miniaturization and high-throughput applications (Table 3). We are aware that many more 3D flow culture systems for hepatocytes have been established, but we focus here on those with the highest potential for acceptance in drug screening processes from our point of view. 

In the multi-compartment hollow-fiber bioreactor, cells attach to the surface of fibers and reorganize themselves to generate a microenvironment close to the physiological *in vivo* situation, while three interwoven hollow fibers ensure the supply with oxygen and nutrients [64]. The original device, which required 800 mL medium and several hundred million cells, has recently been down-scaled to 0.5 mL medium and 20 million cells [65]. Hepatocytes have maintained enzymatic activity and gene expression of CYP1A2, CYP3A4/5, CYP2C9, CYP2D6, CYP2B6, transporters, and phase II enzymes for up to 3 weeks [23,66]. In order to protect the cells from shear stress in the bioreactor, Miranda and co-workers (2010) encapsulated hepatocytes into alginate beads, thereby maintaining hepatocyte functionality for up to one month and enabling continuous sampling without termination of the ongoing experiments.

**Table 3:** Overview of 3D flow culture systems for hepatocytes.

3D System	Cell Type	Observation	Reference	Manufacturer
Hollow fiber bioreactor	Rat hepatocytes Human hepatocytes	Increased albumin synthesis and diclofenac toxicity higher expression of CYP1A2, CYP3A4/5, CYP2C9, CYP2D6, CYP2B6, transporters, and phase II enzymes	[64,66,67,68]	Unisyn
Alginate encapsulated hepatocytes in the bioreactor	Rat hepatocytes	Enhanced biotransformation, CYP inducibility, albumin and urea secretion	[69]	Sartorius Stedim
Multichamber modular bioreactor	Human hepatocytes	Up-regulation of CYP1A1, 1A2, 2B6, 2C9, 3A4, UGT, MDR1, and MRP2	[70]	University of Pisa
Quasi-Vivo^®^	Human hepatocytes	Enables investigation of cross-talk between different cell types	[71]	Kirkstall
LiverChip	Human hepatocytes	Maintained mRNA-levels of Phase I/II-enzymes over 7 days, higher or similar CYP-activities after day 4	[72,73]	CN Bio Innovations Limited
HepaChip^®^	Human hepatocytes	Up-regulation of CYP3A4, CYP2A1, and phase II enzymes	[52]	NMI Reutlingen
3^D^-KITChip	HepG2, rat hepatocytes	Higher level of differentiation	[40]	KIT

Another system applying low shear stress during cultivation is the multichamber modular bioreactor (MCmB). As several silicone culture chambers can be connected to each other, the interaction between the different chambers is solely based on soluble molecules in the cultivation medium, enabling the imitation of exchange between different tissues. However, single chambers can also be used for the cultivation of cells seeded onto scaffolds or embedded in hydrogels. After two weeks of culture, detoxification genes in hepatocytes, such as CYP1A1, 1A2, 2B6, 2C9, 3A4, UGT, MDR1, and MRP2, reached expression levels close to or above those of freshly isolated hepatocytes. Just recently, this cultivation system has been commercialized as the Quasi-Vivo^®^ system (Kirkstall Ltd, Rotherham, UK). Like in other silicon-based systems, one main obstacle is the high substrate adsorption which could lead to an incorrect data interpretation [68].

Without a doubt, the development of these bioreactors has been an important step towards the *in vivo* situation and has improved our knowledge and understanding of hepatocyte biology. Nevertheless, the main disadvantages are the still required high cell numbers, which is in contrast to the restricted availability of these cells [8,16], their limitation to a few laboratories and the required expensive equipment and experienced personnel. Each of these aspects restricts their acceptance in drug research and highlights the demand for simplified systems, which are robust, reproducible, economic, and can easily be adapted to high-throughput screenings for drug testing.

In the LiverChip (Table 3) multi-well platform, developed by Zyoxel (Oxfordshire, UK), cells are seeded onto implemented scaffolds. This platform focuses on three key aspects of hepatic physiology; *i.e.*, the dynamic flow, the organization of cells into structures to mimic the architecture of the sinusoids, and the combined cultivation of hepatocytes and non-parenchymal cells [72]. Primary hepatocytes cultivated under these conditions exhibited a strong *in vitro*-*in vivo* correlation, which indicates a good predictability for hepatic clearance [21], while these cells also showed an *in vivo* like testosterone metabolism [72].

**Figure 1:**
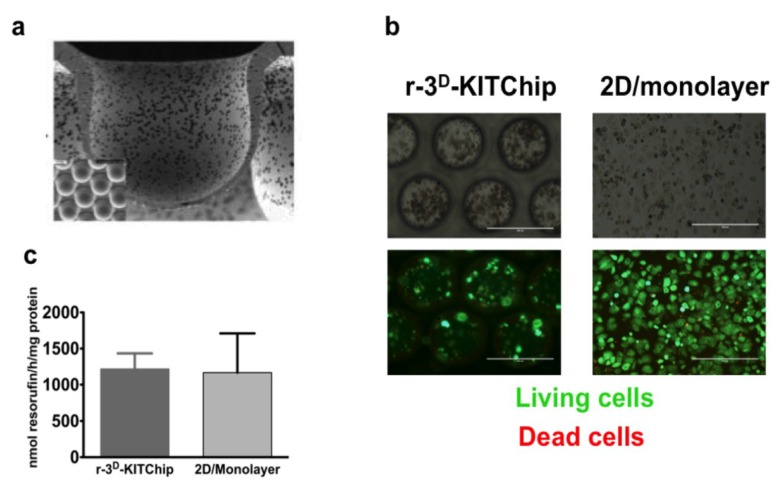
The viability of primary human hepatocytes seeded into the r-3^D^-KITChip. (**a**) Image of the cavities of the r-3^D^-KITChip; (**b**) Bright-field images of primary human hepatocytes seeded into the r-3^D^-KITChip and in 2D (upper part). In the lower part, dead cells have been differentiated from the living cells by staining the living cells with 2 μM Calcein AM, while dead cells were stained with 4 μM ethidium homodimer. Pictures were taken with an Evos*_fl_* microscope from Peqlab Biosystems (Erlangen, Germany). The scale bar corresponds to 400 μm; (**c**) In order to evaluate cell survival of primary human hepatocytes, resazurin conversion into resorufin was measured and normalized to mg protein in comparison to 2D cultivation conditions. The graphic was drawn with the GraphPad Prism software (GraphPad Software, version 5.01, San Diego, CA, USA).

**Figure 2:**
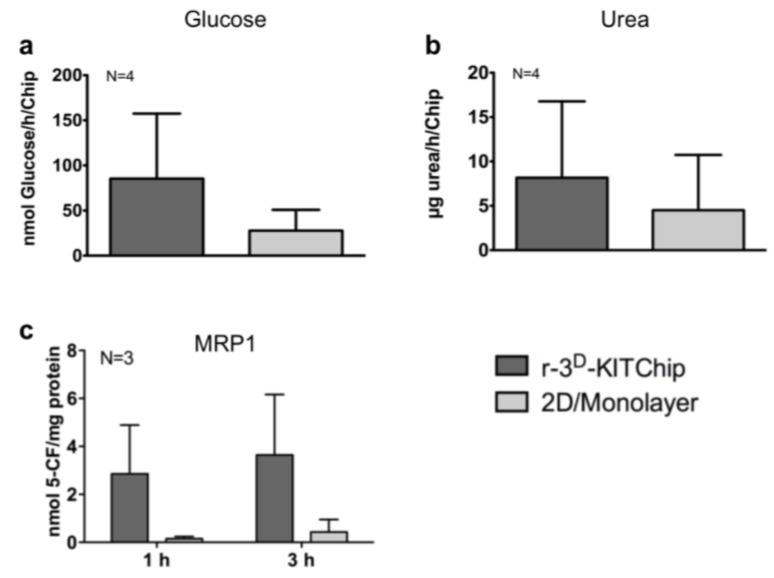
Functional parameters of primary human hepatocytes in the r-3^D^-KITChip and under 2D monolayer conditions on collagen coated cell culture plates. After attachment over night and perfusion for 24 h in the r-3^D^-KITChip, the culture medium was replaced by a buffer containing 1 mM magnesium chloride and 1 mM sodium pyruvate. The perfusion was continued for 24 h and cell culture supernatants were used for measuring basal glucose levels. (**a**) Basal glucose production; (**b**) Urea synthesis; (**c**) multidrug resistant protein 1 (MRP1) activity. Cells were cultivated either in the r-3^D^-KITChip or under 2D conditions in the presence of 5-carboxyfluorescein diacetate (5-CFDA). After uptake, 5-CFDA was metabolized into the fluorophore 5-CF, which was detected in the cell culture supernatants after the indicated time points. The graphs were drawn with the GraphPad Prism software (GraphPad Software, version 5.01). The values are depicted as mean and standard deviation of three donors.

**Figure 3:**
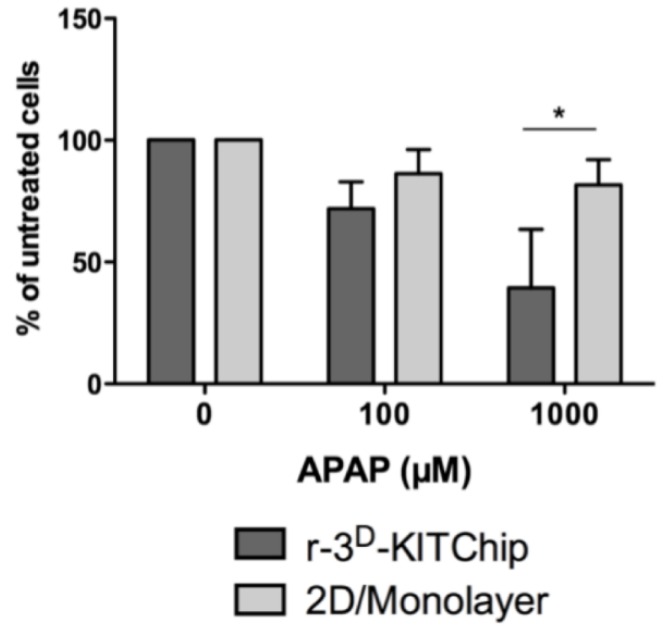
Cultivation in the r-3^D^-KITChip sensitizes primary human hepatocytes to acetaminophen-induced intoxication. The cells were cultivated in the presence of acetaminophen (100 μM and 1000 μM) for 24 h. Their viability was determined by the conversion of resazurin into resorufin and is depicted as % of untreated cells. The graph was drawn with GraphPad Prism software (GraphPad Software, version 5.01). Values are depicted as mean and standard deviation of three donors/experiments.

Another approach, developed by the Karlsruhe Institute of Technology (KIT), is based on a polycarbonate or polymethylacrylate polymer chip [40]. Cells seeded onto the polymer chip have generated three-dimensional structures in the porous microcavities (Figure 1a) of typically 300 µm in each direction, and the human carcinoma cell line HepG2 exhibited a higher expression of uridine-diphosphate-glucosyl-transferase and glutathione-S-transferase. Short-term cultivation of primary human hepatocytes under perfusion conditions (400 μL/h) for 24 h in these polymer chips revealed cell clustering (Figure 1b), while the cells exhibited a good viability (Figure 1c). Furthermore, primary human hepatocytes seeded into the r-3^D^-KITChip demonstrated a higher rate of glucose production (Figure 2a), a higher detoxification rate (Figure 2b), and a higher multidrug resistant protein 1 (MRP1) (Figure 2c) activity than cells cultivated under 2D monolayer conditions. These differences between the two cultivation systems were even more pronounced when primary human hepatocytes were stressed by 100 μM and 1000 μM acetaminophen (Figure 3). Hepatocytes cultivated in the r-3^D^-KITChip under a continuous flow displayed a significantly higher sensitivity towards acetaminophen-induced intoxication, confirming our previously published data on static 3D culture [3].

A recently developed semi-automated system, the so-called HepaChip^®^ is a microfluidic system, which mimics hepatic sinusoids. On the area of a microscopic slide, each one of the eight micro-chambers contains three cell assembly ridges with the length and width of a human liver sinusoid [74]. Two electrodes in each chamber enable accurate cell positioning. In a short-term experiment of about 3 h, hepatocytes cultivated in the HepaChip^®^, had a significant higher activity of CYP3A4, CYP1A2, and phase II enzymes than hepatocytes co-cultivated in 96-well plates in the presence of endothelial cells [74]. The small volume and the low amount of required cells are an advantage of the HepaChip^®^, while its widespread application might be limited by the required sophisticated equipment.

The ibidi^®^ µ-Slide VI ^0.4^ is a commercially available microfluidic platform with six channels which can be perfused in parallel [34]. Its main advantages are the possibility to combine it with different hydrogels and its suitability for live cell imaging. While we have already demonstrated that the functionality of primary human hepatocytes is maintained for up to 28 days under static conditions [34], we wanted to compare the viability of human hepatocytes under static conditions compared with the cultivation in the ibidi^®^ µ-Slide VI ^0.4^ under flow conditions for the duration of one week.

**Figure 4:**
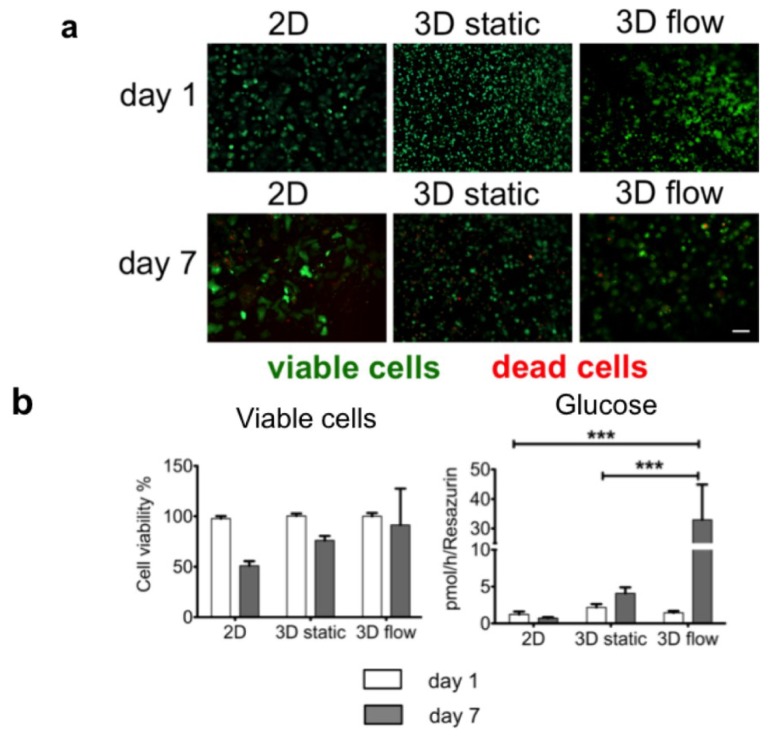
Primary human hepatocytes entrapped in rat-tail collagen combined with continuous flow in the µ-Slide VI ^0^^.4^ show a higher viability and glucose production. Cells were either seeded in 2D or 3D in 96-well plates (2.5 × 10^4^ cells/well) or entrapped in collagen in the µ-Slide VI ^0^^.4^ (1.5 × 10^5^ cells/µ-Slide VI ^0^^.4^) and cultivated for 1 day or 7 days. To evaluate cell viability, the cells were stained either with 2 μM Calcein AM to visualize living cells or with 4 μM ethidium homodimer to visualize dead cells, while the turnover of resazurin to resorufin was measured in parallel. The glucose production was measured from cell culture supernatants. (**a**) Live/dead staining of primary human hepatocytes on day one and day seven. Pictures were taken with an Evos*_fl_* microscope from Peqlab Biosystems (Erlangen, Germany); (**b**) Comparison of resazurin turnover and glucose production. Cell viability is depicted as % of viable cells compared to day one. The graphs were drawn with the GraphPad Prism software (GraphPad Software, version 5.01). The values are depicted as mean and standard deviation of three donors/experiments.

Interestingly, the comparison between the 3D static cultivation and the 3D flow cultivation in the µ-Slide VI ^0.4^ indicated an improved viability and an increased glucose production in the primary hepatocytes exposed to flow for 7 days (Figure 4). These promising first results point out that the exposure to flow under 3D cultivation conditions might improve and maintain hepatocyte function for up to 7 days. In the future, we will investigate these findings further under long-term cultivation conditions. The validation of the µ-Slide VI ^0.4^ for the 3D flow cultivation of human hepatocytes would result in a device with simplified equipment and handling, two main aspects which currently are hindering numerous 3D cultivation systems from high-throughput and broad application in different laboratories and drug testing.

So far, many promising systems for the 3D cultivation of primary human hepatocytes have been developed. As most of these systems have not been tested under uniform conditions, further inter- and intra-laboratory testing on a larger scale is urgently required. Three recent announcements of newly funded projects will hopefully deliver more data in this direction in the near future. Electrospinning and Medicyte have started a co-operation with the objective to characterize the biotransformation of Medicyte’s upcytes^®^ on Alvetex^®^ scaffolds [59]. In a collaboration between InSphero and the group of Ingelman-Sundberg at the Karolinska Insitute (Stockholm, Sweden) the predicitivity of InSphero’s human 3D liver model (3D InSight TM Human Liver Microtissue) will be investigated [75]. Furthermore Promethera will use the CellASIC^®^ microfluidic cell culture platform from EMD Millipore to improve preclinical liver toxicity testing methods [76]. 

The detailed comparison of the generated data in these three projects with existing cultivation systems, like e.g., the 2D conditions, will deliver more reliable data regarding the improvement of the 3D cultivation platforms.

## 4. Conclusions

One of the main objectives concerning the improvement of hepatocyte culture is the maintenance of hepatocyte function. As monolayer cultivation results in a rapid loss of drug-metabolizing enzyme activities, an increasing number of attempts has focused on the cultivation of hepatocytes in different matrices, either naturally derived or based on synthetic scaffolds, in order to mimic a 3D environment.

Recently, numerous attempts have been made to get closer to the *in vivo* situation, as the 3D culture has been combined with perfusion. The main limitation of the first devices was the required number of cells, which is in contrast to the limited availability of primary human hepatocytes. Miniaturization of existing bioreactors through the development of microfluidic devices led to a reduction of the required cell numbers. In order to validate the maintenance of hepatic function in these systems over long-term periods additional data and the direct comparison to the monolayer cultures are required.

Another main obstacle for some of these devices could be the requirement of expensive equipment and their complex handling. For a high acceptance in the drug testing industry and research a simplification of existing devices and their compatibility with high-throughput combined in a user-friendly platform must be achieved.
